# Evaluating Artificial Intelligence (AI)-Generated Patient Education Guides on Epilepsy: A Cross-Sectional Study of ChatGPT and Google Gemini

**DOI:** 10.7759/cureus.73212

**Published:** 2024-11-07

**Authors:** Shiv Arora, Meghna Ramesh, Aye Thandar Moe, Tapan Giri, Kaksha Parrikh, Hima Varsha Challa

**Affiliations:** 1 General Surgery, Sardar Patel Medical College, Bikaner, IND; 2 Internal Medicine, Asian Institute of Gastroenterology Hospitals, Hyderabad, IND; 3 Internal Medicine, University of Medicine 1 Yangon, Myanmar, MMR; 4 Internal Medicine, Byramjee Jeejebhoy Government Medical College, Pune, IND; 5 Medicine, Government Medical College, Bhavnagar, Bhavnagar, IND; 6 Internal Medicine, Sri Ramachandra Institute of Higher Education and Research, Chennai, IND; 7 Internal Medicine, University Hospitals of Leicester NHS Trust, Leicester, GBR

**Keywords:** artificial intelligence, chatgpt, education guide, epilepsy, generalized tonic-clonic seizures (gtcs), google gemini, myoclonic seizures, seizures, status epilepticus

## Abstract

Introduction

Epilepsy is a chronic disorder that requires patient education for management and to avoid triggers and complications. This study aims to evaluate and compare the effectiveness of two artificial intelligence (AI) tools, ChatGPT (version 3.5, OpenAI, Inc., San Francisco, United States) and Google Gemini (version 1.5, Google LLC, Mountain View, California, United States), in generating patient education guides for epilepsy disorders.

Methodology

A patient education guide was generated on ChatGPT and Google Gemini. The study analyzed the sentence count, readability, and ease of understanding using the Flesch-Kincaid calculator, examined similarity using the QuillBot plagiarism tool, and assessed reliability using a modified DISCERN score. Statistical analysis included an unpaired T-test where a P-value <0.05 is considered significant.

Results

There was no statistically significant difference between ChatGPT and Google Gemini in terms of word count (p=0.75), sentence count (p=0.96), average words per sentence (p=0.66), grade level (p=0.67), similarity% (p=0.57), and reliability scores (p=0.42). Ease scores generated by ChatGPT and Google Gemini were 38.6 and 43.6 for generalized tonic-clonic seizures (GTCS), 18.7 and 45.5 for myoclonic seizures, and 22.4 and 55.8 for status epilepticus, respectively, showing Google Gemini generated responses notably better (p=0.0493). The average syllables per word (p=0.035) were appreciably lower for Google Gemini-generated responses, with 1.8 for GTCS and myoclonic, 1.7 for status epilepticus against 1.9 for GTCS, 2 for myoclonic, and 2.1 for status epilepticus for ChatGPT responses.

Conclusions

A significant difference was seen in only two parameters. Further improvement in AI tools is necessary to provide effective guides.

## Introduction

Epilepsy, being one of the most common serious brain conditions, is characterized by the recurrence of unprovoked seizures [1]. It affects over 70 million people worldwide [2]. Patient education in specific seizure types like generalized tonic-clonic seizures (GTCS), myoclonic seizures, and status epilepticus is important as it helps in the early identification of complications and maintenance of medications, thereby preventing complications/early mortality or morbidity. It can also help in the faster resolution of these diseases.

Artificial intelligence (AI) tools have emerged as potential game-changers in patient education, offering readily accessible information on a wide range of diseases. AI also has the potential to revolutionize personalized medicine and enhance population health management. It can also provide virtual health assistants, support mental health care, improve patient education, and influence patient-physician trust [3]. The challenge lies in verifying the accuracy of these tools. There is also a lack of personal connection to doctors. Additionally, there can be an overreliance on these tools for diagnosis and treatment. ChatGPT (version 3.5, OpenAI, Inc., San Francisco, United States) is a revolutionary technology that uses advanced AI techniques to generate natural language responses to a given prompt or input. It has been used across various fields, from natural language processing to customer service to content creation [4]. Google Gemini (version 1.5, Google LLC, Mountain View, California, United States) is a powerful AI model from Google that can understand text, images, videos, and audio. Google Gemini showcases the latest advancements in AI and has attracted attention for its capabilities, which are comparable to the widely popular ChatGPT [5].

The goal is to assess the potential of ChatGPT and Google Gemini to improve the quality and accessibility of patient counseling for individuals living with specific seizure types such as GTCS, myoclonic seizures, and status epilepticus.

Aims and objectives 

To evaluate the effectiveness of ChatGPT and Google Gemini in generating patient education guides for GTCS, myoclonic seizures, and status epilepticus, and to assess the accuracy, comprehensiveness, and readability of AI-generated materials. This manuscript also aims to determine the potential clinical utility of these AI tools in epilepsy patient education and management.

## Materials and methods

A cross-sectional original research study was conducted over one week in June 2024. Since no human participants were involved in this study, ethics committee approval was not needed. 

Firstly, the responses were generated. Three common diseases in neurology were selected, namely GTCS, myoclonic seizures, and status epilepticus. Two AI tools were selected, namely ChatGPT and Google Gemini, for the generation of brochures for patient education. ChatGPT version 3.5 and Google Gemini version 1.5 were used on June 11th, 2024 [6]. The following prompt was given to both AI tools: “Write a patient education guide for [disease name].” Responses generated were collected in a Microsoft Word (Microsoft Corp., Redmond, WA, US) document and were graded using various tools: the Flesch-Kincaid calculator was used for word count, sentence count, ease of understanding and readability of the information generated, similarity of the content was checked using the QuillBot Plagiarism Tool, and reliability of scientific text using a modified DISCERN score [7,8,9]. The modified DISCERN score is a tool to measure reliability and accuracy with a total score of five points. The higher scores indicate higher reliability [9].

Statistical analysis data was exported to a Microsoft Excel sheet. Statistical analysis was done using R version 4.3.2. R core team (2023) (R: a language and environment for statistical computing; R Foundation for Statistical Computing, Vienna, Austria, https://www.R-project.org/). The responses generated by ChatGPT and Google Gemini were compared using an unpaired T-test. A P-value <0.05 was considered significant. The correlation between ease score and reliability score was compared using Pearson’s coefficient of correlation.

## Results

ChatGPT and Google Gemini were used to generate brochures on patient education for GTCS, myoclonic seizures, and status epilepticus.

Table 1 shows that there was no significant difference in the word count (p=0.753), sentence count (p=0.9626), average words per sentence (p=0.6633), grade level (p=0.6714), similarity% (p=0.5722), and reliability scores (p=0.4226) between ChatGPT and Google Gemini.

**Table 1:** Characteristics of responses generated by ChatGPT and Google Gemini + t-test. P-values <0.05 are considered statistically significant.

Variables	ChatGPT		Google Gemini		P-value^+^
	Mean	Standard Deviation	Mean	Standard Deviation	
Words	392.30	128.75	419.70	40.50	0.7543
Sentences	47.00	32.19	48.00	7.81	0.9626
Average words per sentence	10.93	6.74	8.93	1.85	0.6633
Average syllables per word	2.00	0.10	1.77	0.06	0.0357*
Grade level	12.27	2.83	10.90	4.28	0.6714
Ease score	26.57	10.58	48.30	6.56	0.0493*
Similarity %	37.93	34.70	23.80	16.79	0.5722
Reliability score	2.67	0.58	3.00	0.00	0.4226

However, there was a significant difference in the average syllables per word (p=0.0357) and the ease score (p=0.0493) between ChatGPT and Google Gemini. The ease score was significantly higher for Google Gemini-generated responses as compared to ChatGPT, while the average syllables per word were significantly less for Google Gemini-generated responses as compared to ChatGPT.

**Figure 1:**
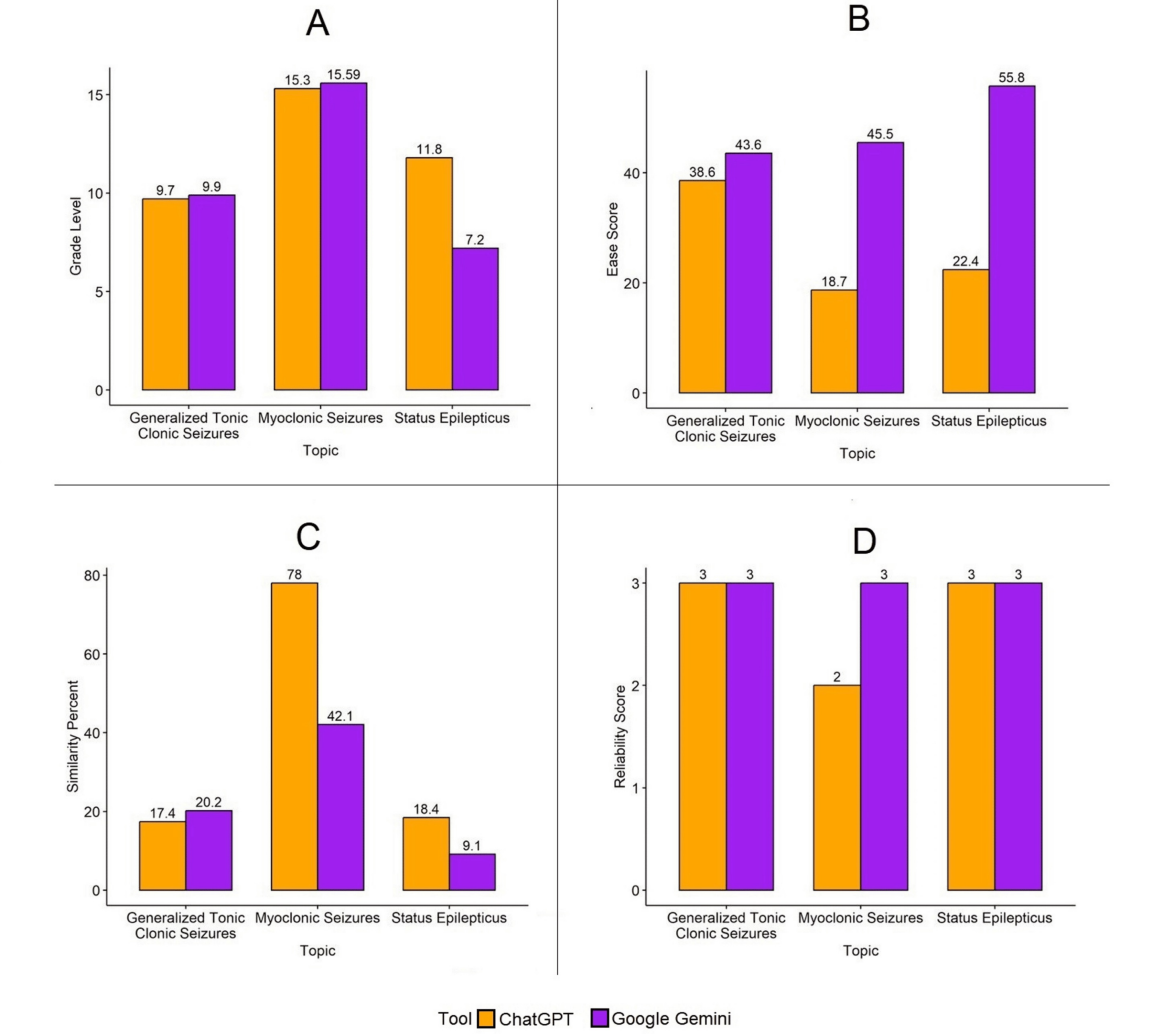
Graphical representation of comparison between grade level, ease score, similarity percent, and reliability score, for the patient education guide generated by ChatGPT and Google Gemini a: comparison of grade level; b: comparison of ease score; c: comparison of similarity%; d: comparison of reliability score

Figure 1 shows that the Flesch-Kincaid grade level of the response generated by Google Gemini was higher (9.9) than ChatGPT (9.7) for the topic of generalized tonic-clonic seizures. Similarly, the Flesch-Kincaid grade level of the response generated by Google Gemini was higher (15.59) as compared to ChatGPT (15.3) for the topic of myoclonic seizures. However, for the topic of status epilepticus, the Flesch-Kincaid grade level of the response generated by ChatGPT (11.8) was higher than Google Gemini (7.2).

The Flesch reading ease score of the patient education brochure generated by Google Gemini on the topic of GTCS was higher (43.8) as compared to ChatGPT (38.6), with both responses having a college reading level (difficult to read). For the topic of myoclonic seizures, the Flesch reading ease score of the response generated by Google Gemini was higher (45.5), corresponding to a college reading level (difficult to read) as compared to the response generated by ChatGPT (18.7), which had a college graduate reading level (very difficult to read).

For the topic of status epilepticus, the patient education brochure generated by Google Gemini had a higher Flesch reading ease score (55.8) with a corresponding reading level of high school (difficult to read) as compared to the response generated by ChatGPT (22.4), which had a college graduate reading level (very difficult to read).

The similarity percentage of the response generated by Google Gemini was higher (20.2%) as compared to ChatGPT (17.4%) for the topic of GTCS. Conversely, for the topic of myoclonic seizures, the similarity percentage of the patient education guide generated by ChatGPT was higher (78%) as compared to Google Gemini (42.1%). Likewise, the similarity percentage of the response generated by ChatGPT was higher (18.4%) as compared to Google Gemini (9.1%) for the topic of status epilepticus.

The modified DISCERN score (reliability score) was the same (three points) for the patient education guides generated by both ChatGPT and Google Gemini on the topics of GTCS and status epilepticus. However, the reliability score was higher for the response generated by Google Gemini (three points) as compared to ChatGPT (two points) for the topic of myoclonic seizures.

## Discussion

A cross-sectional study conducted to compare responses generated by two AI tools, ChatGPT and Google Gemini, for brochures on patient education for GTCS, myoclonic seizures, and status epilepticus revealed that there was no significant difference in the word count, sentence count, average words per sentence, grade level, similarity%, and reliability scores between ChatGPT and Google Gemini. Ease score was higher in Google Gemini as compared to ChatGPT.

AI can play a significant role in a patient's education by providing personalized, accessible, and accurate health information. Its ability to simplify health concerns can make it a powerful tool in the education of patients. Short and concise texts are always simple to read and retain. For easy-to-read brochures, the level of understanding (ease score) should be such that even a high school person can understand. In this study, both ChatGPT and Google Gemini generated responses at the college level of understanding for GTCS. For both myoclonic seizures and status epilepticus, ChatGPT is at a very difficult level, and Google Gemini has college-level difficulty. 

This finding can be compared to a study that used an AI dialogue platform to improve the readability of orthopedic surgery-related education materials to the fifth-grade level and found that it can improve the reading accessibility to orthopedic surgery-related online study material [10]. Another study took 71 articles from patient education materials published by the American Academy of Otolaryngology, and ChatGPT4 was successfully used to translate those materials to sixth-grade reading level [11]. Twenty-one patient education materials on aortic stenosis were successfully used by another study to make it readable at the 10th-12th reading level, highlighting ChatGPT’s role in the future for cardiac health literacy [12]. Another study done in 2016 found that the readability of patient education material from the American Academy of Orthopedic Surgeons (AAOS) has improved from 2008-2016 [13]. A study found AI has revolutionized patient education in dentistry by identifying articles from 2018 to 2024, encompassing empirical evidence and conceptual frameworks related to AI in dental patient engagement and communication [14]. In one study, ChatGPT, Microsoft Copilot (Microsoft Corp., Redmond, WA, US), Google Gemini, and Meta AI (Meta Platforms, Inc., Menlo Park, California, United States) were asked 10 questions regarding cardiac catheterization that were developed by website-based patient education materials. The responses generated had an overall mean reading between the 11th and 13th grade levels of education materials [15]. Thus, both ChatGPT and Google Gemini need to be updated to improve the difficulty level in patient education guides for GTCS, status epilepticus, and myoclonic seizures. 

Similarity percentage relates to copied content from pre-existing literature, and this is used to avoid plagiarism. Plagiarism is detrimental in medical publications as it can be related to the credibility of the author and faith in medical updates. With an increase in medical publications, all need to follow publication ethics [16]. The similarity% of the patient education guide for GTCS was higher in Google Gemini (20.2%) as compared to ChatGPT (17.4%), while ChatGPT has a higher similarity% (78 and 18.4%) than Google Gemini (42.1 and 9.1%) in both myoclonic seizures and status epilepticus, respectively, with the expected similarity limit being 15%, which is only under the limit in the Google Gemini guide for status epilepticus. Patient education articles generated by ChatGPT in common dermatologic conditions have a similarity of 27%, which is also higher than the expected limit [17].

A modified DISCERN score is used for reliability of online/media content which comprises five questions, with each affirmative response being assigned a score of one. In this study, both ChatGPT and Google Gemini received a score of three for content related to GTCS and status epilepticus. For myoclonus seizures, ChatGPT received a score of two, while Google Gemini received a score of three. One similar study on patient education on sports surgery-related diseases by using ChatGPT-4 found that it has the potential to provide quality information with a DISCERN score of 44.75 points [18].

Limitations

This study used only two AI tools. In the future, we need to assess more tools and study more diseases. ChatGPT 3.5 is an older version of artificial intelligence, and it may not provide updated content. Although these educational guides aim to bridge the gap, medical professionals possess the necessary training to ensure patient-centered management, a skill that these AI models lack.

## Conclusions

This study highlights that there is no significant difference in the average ease score, grade score, and reliability score of responses generated by the two AI tools for patient information brochures on GTCS, myoclonic seizures, and status epilepticus. The ease score was higher in Google Gemini between the two software. Similarity% is also higher than the limit of 15% in all education guides except for Google Gemini for status epilepticus.

More advancements or updated AI tools are required to enhance the readability of these neurological conditions.
